# Correction: Cipolletta et al. Risk Perception towards COVID-19: A Systematic Review and Qualitative Synthesis. *Int. J. Environ. Res. Public Health* 2022, *19*, 4649

**DOI:** 10.3390/ijerph20042978

**Published:** 2023-02-08

**Authors:** Sabrina Cipolletta, Gabriela Rios Andreghetti, Giovanna Mioni

**Affiliations:** Department of General Psychology, University of Padua, 35122 Padova, Italy

## Table

In the original publication [1], there was a mistake in Table A1: Comparative appraisal of the studies, regarding the order of the authors. The correct table appears below. The authors apologize for any inconvenience caused and state that the scientific conclusions are unaffected. This correction was approved by the Academic Editor. The original publication has also been updated.

## Author Contributions

The author contributions were updated in the newest version to more clearly depict the contributions made by each author to the article [1]. The modification does not affect in any way the scientific conclusions and was approved by the Academic Editor. The corrected version appears below and the original publication has also been updated.

**Table A1:** Comparative appraisal of the studies.

Authors	Country	Sample	Data Collection Methods	Risk Perception Measures	Key Findings
Germani et al., 2020 [7]	Italy	1045 emerging adults (30% M, 70% F)	Cross-sectional online survey carried out in March	Five-point scale answers to 3 items (Perceived Risk Scale)	High perceived risk scores were reported, and risk awareness was positively correlated with anxiety
Yıldırım and Güler 2021 [8]	Turkey	3109 adults (49.98% M, 50.02% F)	Cross-sectional online survey developed in April	Likert-type five-point scale answers to 8 items (COVID-19 Perceived Risk Scale)	Risk perception presented a significant direct effect on death distress, positivity, and happiness
Faasse and Newby 2020 [9]	Australia	2174 residents (503 M, 1635 F, 36 other)	Cross-sectional online survey developed in March	1 question with a five-point scale answer; 3 questions with Visual Analogue Scale (VAS) answers; 1 question with closed-ended options	Higher perceived personal severity of COVID-19 was a predictor of involvement in protective implementations
Wise et al., 2020 [13]	United States	1591 adults (55% F, 40% M, 5% other)	A combined cross-sectional and longitudinal online survey (both held in March)	VAS-type scale answers	Optimistic bias was observed among participants, and risk perception increased on later dates. Education predicted higher risk perceptions and engagement in precautionary behaviors
Lohiniva et al., 2020 [14]	Finland	116 social media posts and emails from the public	Cross-sectional qualitative data collection done in February	Analysis of social media posts and emails to build a thematic analysis of risk perception	Five different risk perception domains were observed, and people showed low personal control over the situation
Kuang et al., 2020 [15]	India	2044 adults (46% F, 54% M)	Cross-sectional phone call surveys (open-ended questions) were conducted in May	1 open question about perceived personal risk of contracting COVID-19	Low perceived risk of contracting coronavirus was found
Moyce et al., 2021 [16]	United States	20 Latinos living in a rural American community	14 semi-structured interviews with participants over the phone conducted in April	3 open questions	Latinos are less likely to fear the virus because they tend to be more worried with having a pay cut or a job loss
Casanova et al., 2020 [17]	Italy	25 patients receiving treatment, 25 patients that had completed treatment, and 25 healthy peers	A semi-structured online qualitative questionnaire held in March	6 closed-ended questions	The majority presented high risk perceptions and feared for the consequences of being infected with COVID-19
Ilesanmi and Afolabi 2020 [18]	Nigeria	360 adults (62.5% F, 37.5% M)	Cross-sectional interviewer-administered questionnaire driven in June	3 closed-ended questions	The sample presented poor knowledge and low risk awareness towards the new coronavirus
Mouchtouri et al., 2020 [19]	Greece	1858 residents (41.2% M, 58.8% F)	Cross-sectional telephone questionnaire (closed- and open-ended questions) conducted between April and May	Four- or five-point scale type of answers	Most respondents had a sound knowledge of COVID-19, but good practices were not reported on the same level
Asefa et al., 2020 [20]	Ethiopia	416 waiters (191 M, 225 F)	Cross-sectional structured face-to-face questionnaire conducted in June	Likert-type five-point scale answers to 12 items	53.4% of participants presented high risk perception related with being older, knowledge about COVID-19, and partaking preventive behaviors
de Bruin and Bennett 2020 [21]	United States	6684 adults (3226 M, 3458 F)	Extensive cross-sectional online survey carried out in March	2 items with a 0–100% visual linear scale type of answer	Low perceived infection and fatality risks. Perceiving greater risks was linked with implementation of protective behaviors
Duan et al., 2020 [22]	China	3837 adults (1985 M, 1852 F)	Cross-sectional online questionnaire held in February	Five-point scale answers to 3 items	Risk perception was the mediating factor between government intervention and public’s engagement in preventive behaviors
Lee et al., 2021 [23]	South Korea	328 middle school students: 146 boys, 182 girls	Cross-sectional online survey collected from September to October	Likert-type five-point scale answers to 4 items	Risk perception was significantly related to protective behaviors as well as gender and health status
Rivas et al., 2021 [24]	Bolivia	886 Bolivians: 65.1% F, 34.9% M	Cross-sectional online survey carried out during April and May	Likert-type seven-point scale answers to 4 items	COVID-19 information exposure, gender, and adoption of preventive behaviors were positively correlated with risk perception
Savadori and Lauriola 2020 [25]	Italy	572 citizens (54% M, 46% F)	Cross-sectional online survey developed in March	10 items covering risk perception with scale type of answer	Respect for social norms and risk perceptions predicted protective behaviors
Xie et al., 2020 [26]	China	317 adults (48.3% F, 51.7% M)	Cross-sectional online survey conducted in May	Likert-type five-point scale answers to 7 items	Changes in safety behaviors are associated with risk perception and COVID-19 knowledge
Zanin et al., 2020 [27]	Italy	8713 citizens (3490 M, 5223 F). 8282 lived in Italy and 431 abroad	Cross-sectional online questionnaire conducted in March	1 closed-ended question with 4 options	People’s risk perception plays a key role in the adoption of safety actions, in people’s feelings, and in their daily habits
Park et al., 2021 [28]	United States	260 adults (61.77% M, 38.23% F).	Cross-sectional online survey	Likert-type seven-point scale answers to 5 items	OB is negatively related to risk perception, and risk perception increases the use of COVID-19 preventive behaviors
Ahmad et al., 2020 [29]	China	302 participants from 6 Chinese Universities and 2 hospitals (59.93% M, 40.07% F)	Cross-sectional online survey	Likert-type five-point scale answers to 5 items	Government’s guidelines, risk perception, and epidemic knowledge influenced engagement in protective behaviors
Atchison et al., 2021 [30]	United Kingdom	2108 adults (987 M, 1094 F)	Cross-sectional online survey developed in March	Closed-ended questions regarding perceived susceptibility and severity	There was a high engagement in preventive measures correlated with government’s guidance and income
Tomczyk et al., 2020 [31]	Germany	157 adults (80% F, 20% M)	Cross-sectional online survey developed in March	2 items with 0–100% type of answer	Compliance with COVID-19 behavioral recommendations was associated with gender, age, education, and risk perception
McFadden et al., 2020 [32]	United States	718 adults (330 M, 386 F)	Cross-sectional online survey developed in February	Likert-type five-point scale answers to 10 items	Risk perception score was low, and most participants supported the use of restrictive policies for infection prevention
Taghrir et al., 2020 [33]	Iran	240 medical students (98 M, 142 F)	Cross-sectional online survey performed in February	Likert-type four-point scale answers to 2 items	High levels of knowledge and adoption of preventive behaviors were reported as well as moderate risk perceptions. A negative correlation between preventive behaviors and risk perception was present
Mansilla Domínguez et al., 2020 [34]	Spain	16201 adults (51.5% F, 48.5% M)	Cross-sectional online survey was conducted for 5 consecutive days	59 items divided in 4 content areas (including risk perception) and different answer types	Gender, age, direct contact with the virus, employment, and health perception were associated with risk awareness
Mora-Rodríguez and Melero-López 2021 [35]	Spain	2034 citizens (52% F, 48% M)	Cross-sectional online questionnaire carried out in March	Likert-type five-point scale answers to 4 items	Greater exposure to COVID-19 news increased personal risk perception. Being older and female predicted higher risk awareness
Roupa et al., 2021 [36]	Cyprus	494 Healthcare workers (HCW) (66.7% F, 33.3% M) and nurses (75.4%)	Cross-sectional online questionnaire that took place in May	Likert-type four-point scale answers to 5 items	No significant correlation was found between COVID-19 perceptions and knowledge
Iorfa et al., 2020 [37]	Nigeria	1554 adults (42.7% F, 57.3% M)	Cross-sectional online survey developed in April	Likert-type seven-point scale answers to 9 items	Risk perception mediates the link between COVID-19 knowledge and adoption of preventive behaviors. Age and gender influence this adoption
Lanciano et al., 2020 [38]	Italy	980 adults (544 F, 436 M)	Cross-sectional online survey conducted in April	Ten-point scale answers to 11 items	Financial and work risk perceptions were higher than the health one. Involvement in preventive measures was related with age, gender, and education
Ciancio et al., 2020 [39]	United States	5414 adults	Cross-sectional online survey that was driven in March	4 items with 0–100 scales	An overestimated mortality risk was observed; risk perception was related to age, education, sources of news, and location
Germani et al., 2020 [40]	Italy	1011 emerging adults (291 M, 720 F)	Cross-sectional online survey developed in March	Five-point scale answers to 3 risk perception dimensions: general/social, self/personal, and relatives/others	Participants showed a higher risk tolerance for their relatives than for themselves
Ding et al., 2020 [41]	China	1461 college students (639 M, 822 F)	Cross-sectional online survey conducted in February	Five-point scale answers to 4 items	Chinese college students expressed high risk awareness (especially females and the ones located in the Hubei area)
Yang et al., 2020 [42]	Canada	3037 adolescents and young adults from Quebec (74.6% F, 25.4% M)	Cross-sectional online survey collected in April	11-point VAS scale answers to 2 items	Factors associated with higher risk perception include higher disease knowledge, presence of a chronic disease, and partaking in precautionary behaviors
Kabito et al., 2020 [43]	Ethiopia	623 residents (402 F, 221 M)	Cross-sectional face-to-face structured questionnaires conducted in April	Five-point scale answers to 5 items	Participants showed low levels of risk perceptions. Age, education, and knowledge were associated with risk awareness
Harapan et al., 2020 [44]	Indonesia	1379 adults (65.7% F, 34.3% M)	Cross-sectional online questionnaire driven between March and April	1 item with 0–100% type of answer	High risk perception was linked with age, income, being unmarried, living area, and profession. Participants showed low risk awareness
Dryhurst et al., 2020 [45]	Australia, Germany, Italy, Japan, South Korea, Spain, Sweden, UK, and USA	6991 participants	Cross-sectional online survey (data were collected between March and April)	Likert-type seven-point/five-point scale answers to 6 items	Levels of concern are higher in the UK, and being male was associated with lower perceived risk
Jahangiry et al., 2020 [46]	Iran	3727 adults (1933 F, 1794 M)	Cross-sectional online survey carried out between March and April	Likert-type five-point scale answers to 8 items	56.4% of participants were implementing preventive behaviors
Abir et al., 2020 [47]	Bangladesh	Two samples (N1 = 322 and N2 = 683)	Two cross-sectional online surveys (one conducted in March and the other in May)	Likert-type five-point scale answers to 5 items	Low risk perception was associated with gender and education. Perceived risk scores decreased between early and late lockdown
Karasneh et al., 2021 [48]	Jordan	486 pharmacists (382 F, 104 M)	Cross-sectional online questionnaire	Likert-type three-point scale answers	Risk was highly perceived among participants, and it was predicted by gender and location. Media use influenced risk awareness
Wang et al., 2020 [49]	China	2058 participants (54.2% F, 45.8% M)	Cross-sectional online survey developed in March	Closed-ended questions	Most participants stated they would get vaccinated in the future. This was related with gender, being married, and high-risk perceptions
Karout et al., 2020 [50]	United States	410 Latino participants (65.9% F, 34.1% M)	Cross-sectional semi-structured questionnaire/interview collected between July and August	Three-point scale answers to 9 items	Low risk perception scores and low engagement in preventive behaviors were found among respondents
Chou et al., 2020 [51]	China (Taiwan region)	1954 adults (649 M, 1305 F; 640 HCW and 1314 members of the general public)	Cross-sectional online questionnaire developed in April	Five-item questionnaire with answers in scale-type	Healthcare professionals had a higher coronavirus risk perception and adopted more protective behaviors than the general public
Peres et al., 2020 [52]	Portugal	3403 residents (2672 F, 731 M). HCW = 545	Cross-sectional online questionnaire conducted in March	Likert-scale type of answers to 6 items	Healthcare workers presented higher COVID-19 risk perception scores than the general population
Gorini et al., 2020 [53]	Italy	650 HCW (439 F, 211 M) from two hospitals in Lombardy	Cross-sectional online questionnaire conducted in May	Slider-scale type of answer (0–100) to 4 items	Healthcare professionals believed they were more at risk for contracting COVID-19 than their family members. Nurses showed the highest risk perception scores
González et al., 2021 [54]	Spain	557 nurses from 26 different public hospitals in Madrid (87.4% F, 12.6% M)	Cross-sectional online questionnaire collected in April	Likert-type four-point scale answers to 4 items	37.5% of nurses were afraid of becoming infected and its consequences, and 62.8% were concerned about infecting their relatives
Niepel et al., 2020 [55]	United States	Two samples (N1 = 1182 and N2 = 953)	Cross-sectional online survey done in March and repeated in April	9-point scale (0–75%)	There was a low perceived fatality risk among participants, but the numbers increased in the second survey done
Tran and Ravaud 2020 [56]	France	7169 participants (5616 F, 1553 M) with chronic conditions	Cross-sectional online survey collected between March and April	1 question with yes/no type of answer	63% of the patients felt at risk of presenting severe illness if contracting COVID-19 because of their condition
Heydari et al., 2021 [57]	Iran	3213 adults (1591 M, 1620 F)	Cross-sectional online survey performed in March	Likert-type five-point scale answers to 4 items	Risk perception mediates the relationship between risk communication and preventive behaviors
Seale et al., 2020 [58]	Australia	1420 adults (740 F, 680 M)	Cross-sectional online survey carried out in March	Likert-type five-point scale answers to 10 items	Low risk perception scores were informed, and adopting preventive behaviors was associated with government trust
Vai et al., 2020 [59]	Italy	2223 adults (675 M, 1548 F)	Cross-sectional online survey conducted between February and March	2 questions with scale-type of answers	Attitude to vaccinate and utility of prevention behaviors were associated to COVID-19 risk perception and media use
Nazione et al., 2021 [60]	United States	698 adults (53.7% F, 45.1% M, and 0.9% other)	Cross-sectional online survey collected in March	8 items with closed-ended questions	Information exposure was not related with COVID-19 risk perception
Capone et al., 2020 [61]	Italy	1124 University students (79.6% F, 20.4% M)	Cross-sectional online questionnaire performed in March	Likert-type seven-point scale answers to 2 items	University students presenting high levels of information seeking also showed higher levels of wellbeing and risk perception
Huang and Yang 2020 [62]	United States	381 adults (58% M, 42% F)	A two-wave, cross-sectional online survey design conducted in April	Likert-type five-point scale answers to 2 items	Risk perception and uncertainty promote information seeking
Jiang 2020 [63]	China	472 Chinese students (227 M, 245 F)	Cross-sectional online survey collected in February	90-item symptom checklist scale with Likert-type five-point answers	56% of students had sufficient knowledge of COVID-19 typical symptoms, and 57% of them reported high risk perception
Soni et al., 2021 [64]	India	217 Delhi adults (116 F, 101 M)	Cross-sectional online survey opened between April and May	Five-point Likert scale answers to 6 items	Having knowledge about COVID-19 is essential to change someone’s perception and attitudes towards it
Geldsetzer 2020 [65]	USA and the UK	2986 adults residing in the USA and 2988 in the UK	Cross-sectional online survey collected in February	0–100% type of answers	The general public held several misconceptions regarding COVID-19
Gollust et al., 2020 [66]	United States	1007 American adults (62.6% were white, 12% Black, 16.5% Hispanic, and 8.9% other)	Cross-sectional online survey done in April	4 items with closed-ended questions	Perceptions of mortality disparities were found among health status and age but not race or finances
Ding et al., 2020 [67]	China	1081 adults (38.85% M, 61.15% F)	Cross-sectional online survey implemented in February	Five-point scale answers to 14 items	Risk perception strongly affects the public’s mental health.
Krok and Zarzycka 2020 [68]	Poland	226 HCW (58.8% F, 41.2% M)	Cross-sectional online questionnaire held between March and May	Five-point scale answers to 18 items	Risk awareness is negatively related to psychological well-being and increases coping strategies
Liu et al., 2020 [69]	China	4991 adults (2514 F, 2477 M)	Cross-sectional online survey held in February	Five-point Likert scale answers to 2 items	Respondents reported low-to-medium levels of risk perception, and high risk awareness was linked to more anxiety
Orte et al., 2020 [70]	Spain	806 adults (248 M, 556 F, 1 other)	Cross-sectional online survey conducted in March	Five-point Likert scale answers to 17 items	There was a positive correlation between distress and COVID-19 risk perception
Qian and Li 2020 [71]	China	351 adults (162 M, 189 F)	Cross-sectional online survey collected in February	2 closed-ended questions	Risk event involvement was positively related to COVID-19 risk perception as well as anxiety
Spinelli et al., 2020 [72]	Italy	854 parents of children aged between 2 and 14 years old (797 F, 57 M)	Cross-sectional online survey conducted in April	Scale-type of answers	Parents’ perceptions of the COVID-19 situation are deeply linked with parents’ stress levels and children’s psychological disturbances
Li et al., 2021 [73]	China (Taiwan region)	1970 adults (1305 F, 650 M, 15 transgender)	Cross-sectional online survey completed in April	5 questions with scale-type of answers	High risk perceptions mediated the association between lower perceived support and higher active coping with COVID-19
de Bruin et al., 2020 [74]	United States	5517 adults (48% M, 52% F; 37% Democrats, 32% Republicans, and 31% other)	Cross-sectional online survey that was driven in March	4 questions with VAS-scale type of answers	Democrats showed higher perceived risk scores and likelihood to engage in preventive behaviors than Republicans
Lachlan et al., 2021 [75]	United States	5000 residents (2435 M, 2558 F, 25 other, and 1 did not answer)	Cross-sectional online survey developed between April and June	Event Hazard/Outrage scale (32 items)	Risk perceptions may vary across preferences for conservative or liberal bias, but there are no differences in mitigation behavior across patterns of media use
Siegrist et al., 2021 [76]	Switzerland	1585 citizens from the German-speaking part (50.9% F, 49.1% M)	Cross-sectional online survey that was driven between March and April	Seven-point scale type of answers to 7 items	People with high general trust perceive less risks associated with COVID-19 but not the ones with high social trust
Ye and Lyu 2020 [77]	China	11783 adults	Cross-sectional online survey	Chinese General Social Survey	Social trust is linked to a higher risk perception and a lower infection rate, and generalized trust is the opposite
Zajenkowski et al., 2020 [78]	Poland	263 adults (27.8% M, 71.5% F, 0.8% other)	Cross-sectional online survey collected in April	Situational Eight Diamonds Scale (40 items) with seven-point scale answers	Grave situations (like the coronavirus pandemic) leave less room for personality traits in predicting behaviors because they overpower dispositional tendencies
Marinthe et al., 2020 [79]	France	Two samples (N1 = 762 and N2 = 229)	Two cross-sectional online questionnaires. The first was conducted in early March and the second in later March	Perceived risk of contamination of the French population, personal contamination, and death were measured by single items (percentage)	Conspiracy was associated with a higher perceived COVID-19 risk of death but not with other risks
Monzani et al., 2021 [80]	Italy	414 adults (70.3% F, 29.7% M).	Cross-sectional online questionnaire completed by participants between March and April	0–100 slider scale answers to 8 items	People presenting more dispositional optimism indicated elevated levels of optimistic bias
Puci et al., 2020 [81]	Italy	2078 HCW (78.8% F, 21.2% M)	Cross-sectional online survey developed from May to June	Five-point scale answers to 7 items	The majority presented high risk infection perceptions (especially nurses and physicians)
Ferdous et al., 2020 [82]	Bangladesh	2017 residents (59.8% M, 40.2% F)	Cross-sectional online survey conducted between March and April	Closed-ended questions to 4 items	Participants showed a high COVID-19 risk perception and high partaking in safety behaviors
Serwaa et al., 2020 [83]	Ghana	350 adults (56% M, 44% F)	Cross-sectional online questionnaire collected in March	3 closed-ended questions	Participants had a good COVID-19 knowledge and high risk awareness
Samadipour et al., 2020 [84]	Iran	364 adults (154 M, 201 F, and 9 did not answer)	Cross-sectional online survey conducted between February and March	Five-point Likert scale answers to 26 items	Iranians have a moderate risk perception of COVID-19. Five factors contribute to it: cultural, political, emotional, cognitive, and social
Shiina et al., 2020 [85]	UK, Spain, and Japan	4000 people from Japan, 2000 from the UK, and 2000 from Spain	Cross-sectional online survey. Data were gathered between March and April	Nine-point scale type of answers	Knowledge, anxiety, and the frequency of precautionary behaviors was higher in the UK and Spain than in Japan
Soiné et al., 2021 [86]	Germany	Young adults (24–26 y) that belong to different ethnic groups	Data from the CILS4COVID survey were used	2 closed-ended questions comparing financial and health risk perceptions	Ethnic minorities show more health and financial risk perceptions than the general population
